# Supplementary material for: An Approach to Evaluate the Effective Cytoplasmic Concentration of Bioactive Agents Interacting with a Selected Intracellular Target Protein
Source: Pharmaceutics. 2023 Jan 18;15(2):324. doi: 10.3390/pharmaceutics15020324 (PMC9965106; doi:10.3390/pharmaceutics15020324)
Supplement: Supplementary file 1 [file pharmaceutics-15-00324-s001.zip › pharmaceutics-2000204-supplementary.pdf]

Supplements

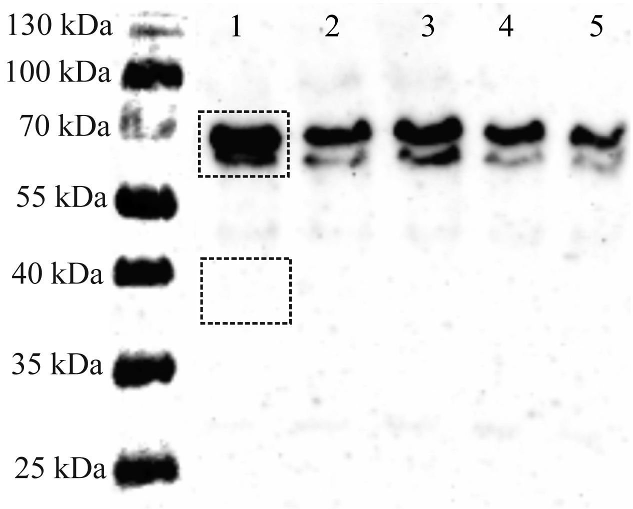

Figure S1. Western blot with Keap1 antibodies for 3LL cell lysate. The dotted square shows the area where the intensities for the studied sample and for the background were determined. The samples from left to right: unheated (band 1), heated to temperatures of 42°C (band 2), 44°C (band 3), 46°C (band 4) and 48°C (band 5) for 3 min.

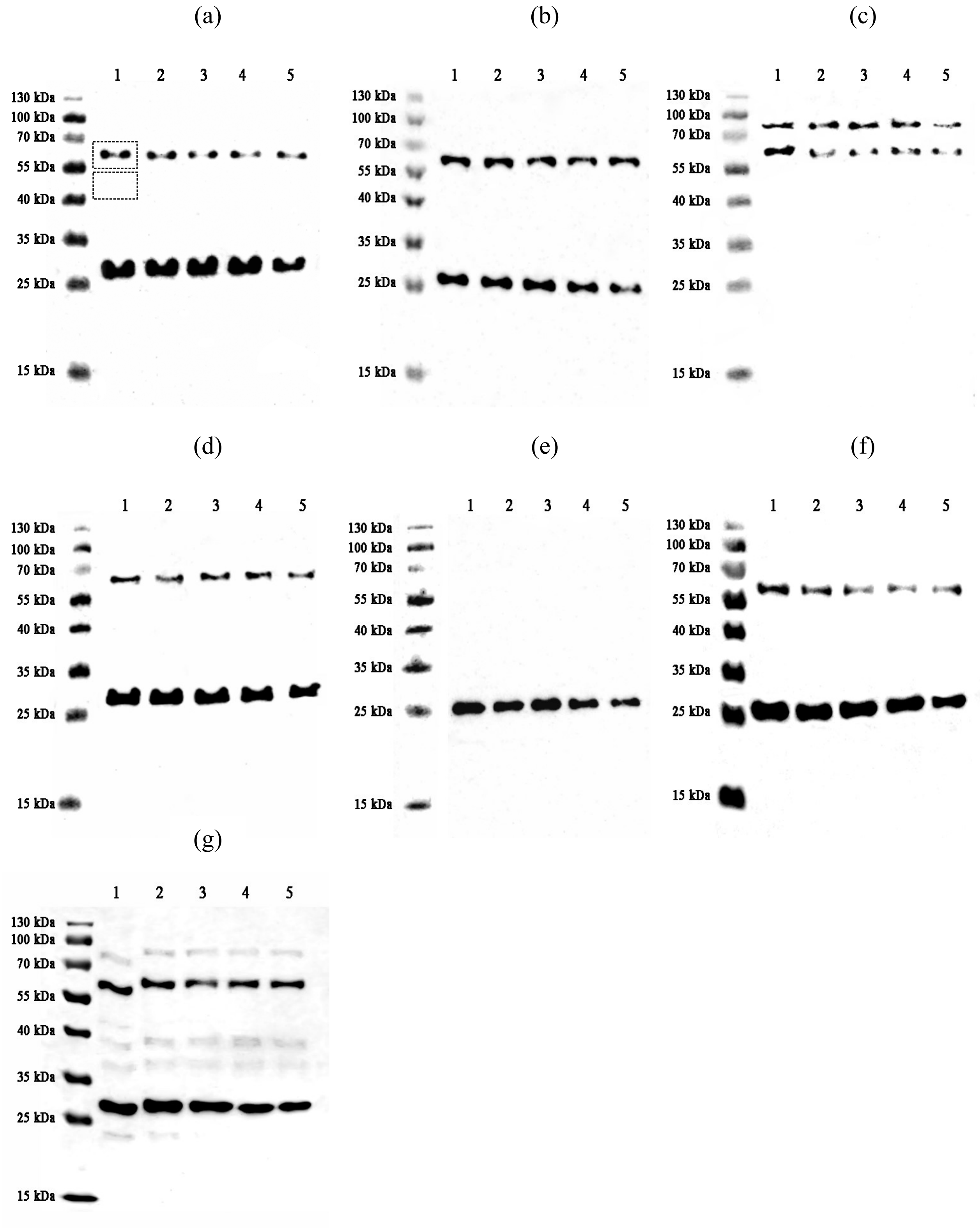

Figure S2. Western blot with Nrf2 antibodies for RAW264.7 (a), Hepa-1 (b), MEF (c), AML12 (d) and 3LL (e) cell lysate, 3LL cell lysate in different cocktail of inhibitors (f) and 3LL cell lysate with MG-132 (g). The dotted line shows the area in which the intensities for the studied sample and for the background were determined. The samples from left to right: unheated (band 1), heated to temperatures of 42°C (band 2), 44°C (band 3), 46°C (band 4) and 48°C (band 5) for 3 min.

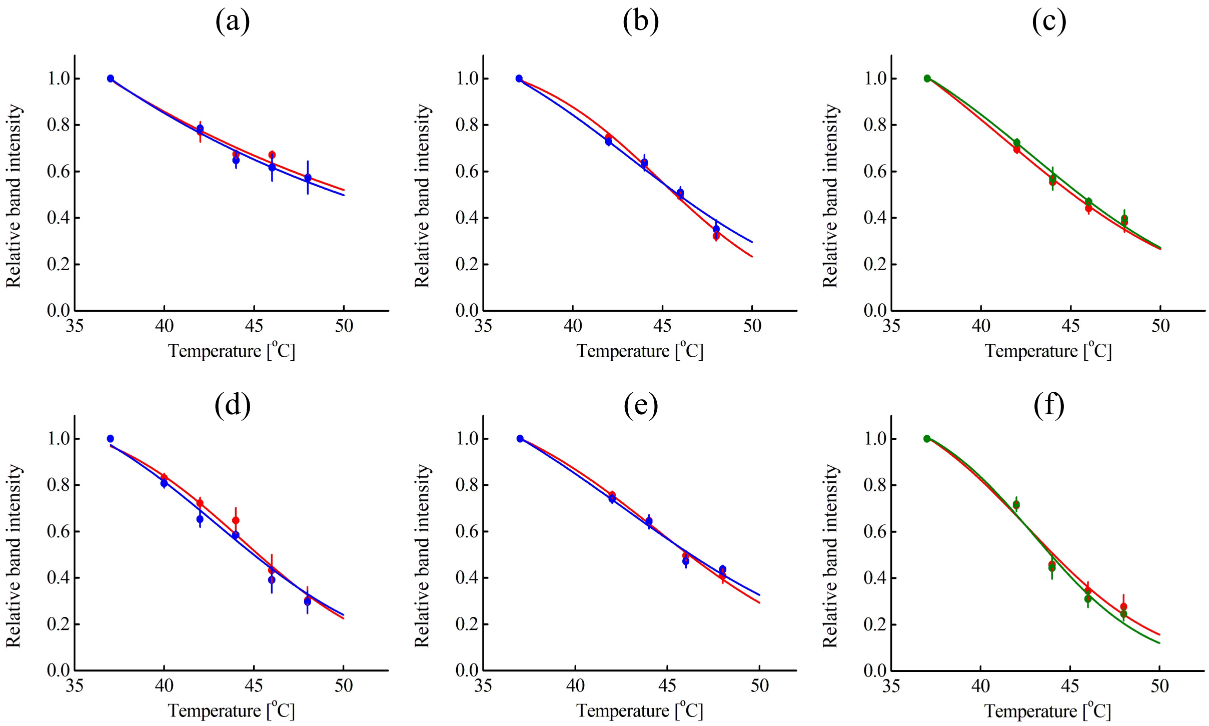

Figure S3. Melting curves of Nrf2 for RAW264.7 (a), Hepa-1 (b), MEF (c), AML12 (d), 3LL (e) and 3LL with MG-132 (f) cell lysate obtained from a band of about 65 kDa (red curve), about 28 kDa (blue curve) and about 85 kDa (green curve). SE are shown (n=4).
